# Assessing mycoplasma contamination of cell cultures by qPCR using a set of universal primer pairs targeting a 1.5 kb fragment of 16S rRNA genes

**DOI:** 10.1371/journal.pone.0172358

**Published:** 2017-02-22

**Authors:** Audrey Jean, Florence Tardy, Omran Allatif, Isabelle Grosjean, Bariza Blanquier, Denis Gerlier

**Affiliations:** 1 Univ Lyon, SFR BioSciences, ENS de Lyon, Inserm US8, CNRS UMS344, UCBL, Lyon, France; 2 ANSES, Agence Nationale de Sécurité Sanitaire de l’Alimentation, de l’Environnement et du Travail, VetAgro Sup, Univ Lyon, UMR Mycoplasmoses des Ruminants, Marcy l'Etoile, France; 3 CIRI, International Center for Infectiology Research, Inserm, U1111, Université Claude Bernard Lyon 1, CNRS, UMR5308, Ecole Normale Supérieure de Lyon, Univ Lyon, Lyon, France; The Ohio State University, UNITED STATES

## Abstract

Mycoplasmas (a generic name for *Mollicutes*) are a predominant bacterial contaminant of cell culture and cell derived products including viruses. This prokaryote class is characterized by very small size and lack of a cell wall. Consequently, mycoplasmas escape ultrafiltration and visualization under routine microscopic examination, hence the ease with which cells in culture can be contaminated, with routinely more than 10% of cell lines being contaminated. Mycoplasma are a formidable threat both in fundamental research by perverting a whole range of cell properties and functions and in the pharmacological use of cells and cell derived products. Although many methods have been developed, there is still a need for a sensitive, universal assay. Here is reported the development and validation of a quantitative polymerase chain reaction (qPCR) based on the amplification of a 1.5 kb fragment covering the 16S rDNA of the *Mollicute* class by real-time PCR using universal U1 and U8 degenerate primers. The method includes the addition of a DNA loading probe to each sample to monitor DNA extraction and the absence of PCR inhibitors in the extracted DNA, a positive mycoplasma 16S rDNA traceable reference sample to exclude any accidental contamination of an unknown sample with this reference DNA, an analysis procedure based on the examination of the melting curve and the size of the PCR amplicon, followed by quantification of the number of 16S rDNA copies (with a lower limit of 19 copies) when relevant, and, if useful, the identification of the contaminating prokaryote by sequencing. The method was validated on a collection of mycoplasma strains and by testing over 100 samples of unknown contamination status including stocks of viruses requiring biosafety level 2, 3 or 4 containments. When compared to four established methods, the m16S_qPCR technique exhibits the highest sensitivity in detecting mycoplasma contamination.

## Introduction

Cells lines and primary cell cultures are very frequently used as tools to unravel the molecular and cellular mechanisms that underlie biological processes, such as cell-invasion by viruses, microbes or parasites. In order to avoid biased interpretation of cell-based experiments, these tools should be kept under stringent quality scrutiny. Together with errors in cell line authentication, contamination by mycoplasmas is recognized as one of the two major pitfalls in cell culture. To give just one example, a survey of transcriptomic data deposited in NCBI Sequence Read Archive has shown that more than ten percent of the samples contained identifiable RNA from mycoplasmas meaning that many transcriptomic data have been published though being invalid [1]. Mycoplasmas are wall-less prokaryotes of very small size with a DNA genome in the Mb range. They belong to the *Mycoplasmataceae* family, *Mycoplasmatales* order, *Mollicutes* class and *Tenericutes* division. Their small size favours their unnoticed cohabitation with culture cells and their plasticity allows them to pass through 0.2 μm filters. Mycoplasma growth impedes many functions of eukaryotic cells with dreadful perturbation of data obtained in culture systems. They induce a cellular reprogramming of the transcriptome [1], change cellular metabolism, affect signal transduction, cell growth or apoptosis, DNA and RNA synthesis, all of these leading to perverted data during biochemical and biological assays. Furthermore, virus growth can be either favoured or disfavoured [2]. In nature, mycoplasma species can be either commensals or pathogens to humans, animals, and plants.

Detecting a mycoplasma contamination is not straightforward. Gold standards look for growth of mycoplasma colonies cultured on broth agar over several weeks and search for extra-nuclear DNA dots stained with Hoechst’s reagent. In both cases, this means days or weeks of culture to allow the growth of the mycoplasma until the colonies reach a size large enough to be seen macroscopically and microscopically, respectively. Other techniques have been developed, such as enzymatic- and bio-assays, ELISA, and polymerase chain reaction (PCR). These techniques are either cumbersome, difficult to interpret, of limited sensitivity and/or limited to the detection of only a limited range of species [3, 4]. Furthermore their use to detect mycoplasma contamination in virus stocks can be difficult or even impossible for highly pathogenic viruses that have to be manipulated in biosecurity level (BSL) 3 or 4 containments. Here is reported a highly sensitive quantitative or real-time PCR termed m16S_qPCR. It is based on the selective amplification of a 1.5 kilobase DNA fragment using universal degenerate U1/U8 primers that target the mycoplasma 16S rDNA [5]. In addition, it can be followed by a sequencing-based identification step. To validate the m16S_qPCR, hundreds of samples from either cell culture or BSL2 to BSL4 viral stocks were tested for mycoplasma contamination by this technique and whenever possible, compared with four other assays—Hoechst DNA staining, MycoAlert and PlasmoTest and PCR. The limitations that have been found with the last four techniques and the detection of a case of contamination by a very unusual mycoplasma strain using m16S_qPCR are also reported.

## Materials and methods

### Sample preparation and biosafety

Supernatants of cell culture were prepared by harvesting cell free supernatants and further clearance of cell debris by centrifugation in 15 mL conical tubes at 200 g at room temperature for 5 minutes. They were kept frozen at -80°C until use. All samples were manipulated under a Type II laminar flow and biosecurity level laboratory containment (BSL2, 3 or 4) as required for the manipulation of cells and viruses until their full inactivation. Samples include routine checking for mycoplasma contamination in cell lines and virus stocks (or infected cells) of RNA viruses (measles, canine distemper, vesicular stomatitis, Ebola, Nipah, influenza, Crimean-Congo haemorrhagic fever, human T lymphotropic I, Drosophila C, Drosophila X, Mopeia, Puumala, Gypsy virus) and DNA viruses (Epstein Barr, BK virus).

### DNA staining by Hoechst reagent

The principle is to detect mycoplasma colonies growing adjacent to cells by visualizing DNA dots located outside the cell nuclei. The indirect assay was used as described previously [3]. Briefly, a suspension of indicator cells was prepared by detachment from the tissue culture flask by a short trypsin-EDTA treatment, counting and resuspension into fresh tissue culture medium. To include suitable cell lines that can resist the cytopathic effect of several human viruses that our collaborators were working with, the assay was validated with five cell lines of different species origin (see Table 1 for details). Square (22 x 22 mm) sterile glass coverslips were deposited at the bottom of a 6-well microplate. Two mL of indicator cell suspension per well were allowed to adhere overnight before the addition of 0.2 mL of the cell-free sample to be tested. Cells were left to grow over 5–7 days in 5% CO_2_ in humidified atmosphere at 37°C. When cells reach ~50–80% confluence, the supernatant was carefully discarded, the cell monolayer was carefully washed once with PBS1X pH 7.2 and fixed for 10 min at RT by adding 2 mL of Ethanol/Acetic acid (3:1, vol:vol). The fixative was discarded and 2 mL of 2 μg/mL of Hoechst (bis Benzimide, Sigma cat. no. B-2883) solution diluted in PBS1X from a 500X stock solution kept aliquoted at -20°C, was added and incubated for 10 min at room temperature. The staining solution was discarded, and followed by two careful washes with clear water. After addition of 15 μL of FluoPrep (BioMérieux, cat.n. 75521), the coverslip was transferred onto a glass slide so as to have the cell monolayer in contact with the slide. The slide was examined by UV epifluorescence microscopy (360 nm excitation filter and barrier filter allowing 490–500 nm emission, 100X oil objective). Multiple fields were recorded for the absence or presence of fluorescent dots. Biohazard: paraformaldehyde and Hoechst solutions and UV microscope are hazardous. Since the adaption of this procedure by using Ibidi polymer coverslips (Biovalley, cat n. 80826, n. 80446) instead of glass coverslips was unsuccessful, the Hoechst staining assay could not be used in either BSL3 or BSL4 containment according to the local biosafety regulations.

**Table 1:** Indicator cell lines for mycoplasma detection using indirect Hoechst’ staining.

Name	Species	Culture medium^1^	cells/well^2^
MeWo [6]	Human	RPMI 1640 Glutamax-I, 10% fetal calf serum, 1% Sodium pyruvate, 1% non-essential amino acids, 10 mM HEPES pH7.2	4x10^4^
Vero [7]	Monkey	DMEM GlutaMax-I,10% fetal calf serum	8x10^3^
IgH-2 [8]	Iguana	EMEM, 2 mM Glutamax, 10% fetal calf serum 1% non-essential amino acids	6x10^3^
NIH3T3 [9]	Mouse	RPMI 1640 Glutamax-I, 5% fetal calf serum	4x10^3^
CHO [10]	Hamster	Ham’s F-12 Nutrient Mixture, 10% fetal calf serum	4x10^3^

^1^ in humidified incubator, in the presence of 5% CO2 at 37°C

^2^ amount of cells to be seeded in 2 mL of culture medium in 6 well-plates.

## Detection of mycoplasma specific enzyme using MycoAlert

The principle is to detect a mycoplasma specific enzyme using a luciferase-based assay. The procedure was performed as recommended by the manufacturer (http://bio.lonza.com/uploads/tx_mwaxmarketingmaterial/Lonza_ManualsProductInstructions_MycoAlert_Mycoplasma_Detection_Kit.pdf). Critical step: samples should not be heated to avoid destruction of the mycoplasma enzyme. The reagent mixture should be vortexed and centrifuged to ensure homogeneity. Data were expressed as either below or above the 50 colony forming unit (CFU) threshold as defined by the manufacturer.

### Detection of mycoplasma lipopeptides using PlasmoTest

The principle is to detect mycoplasma lipopeptides by Toll like receptor 2 (TLR2). This requires the use of a HEK-Blue-2 reporter cell line that stably expresses TLR2, which upon binding to a lipopeptide agonist activates the secretion of alkaline phosphatase. The secreted enzyme is detected by colorimetry. The procedure was performed as recommended by the manufacturer (http://www.invivogen.com/PDF/PlasmoTest_rep_pt1_TDS.pdf). HEK-Blue-2 cells should be used at least 2 days after passaging and before reaching 80% confluency.

### Plasmid DNAs used as controls for m16S_qPCR

The p_GFP plasmid coding for the green fluorescent protein described elsewhere [11] was used as a DNA loading tracer during DNA purification.

The 1514 bp 16S rDNA fragment from *M*. *capricolum* subsp. *capricolum* strain California Kid (gi_83283139) was subcloned using U1 and U8 degenerate primers (Table 2) [5] in the EcoR V restriction site of pBSK(+) to give p_m16S(1.5kb). This plasmid was further digested by Eco47III to delete a 601 bp internal fragment to give p_m16S(0.9kb). The 16S rDNA insert was fully sequenced in both plasmids. The p_m16S(0.9kb) plasmid was used as the positive reference DNA for m16S_qPCR because it can be traced as a possible accidental contaminant by determining the much shorter amplicon size it will give.

**Table 2:** Sequences and optimal amounts of GFP and mycoplasma 16S primers for their use in real-time PCR.

Primer name	Primer sequence*	Final concentration	Reference
16S U1 (forward)	5’–GTTTGATCCTGGCTCAGGAYDAAC– 3’	1 μM**	[5]
16S U8 (reverse)	5’–GAAAGGAGGT**RW**TCCA**Y**CC**S**CAC– 3’	2 μM***	[5]
GFP_for	5’–ATGGTGAGCAAGGGCGAGGA– 3’	0.2 μM	[12]
GFP_rev	5’–CTCGCCGGACACGCTGAACT– 3’	0.2 μM	[12]

* **Y** = C or T; **D** = T, A or G; **R** = A or G; **W** = A or T; **S** = C or G.

** or 0.17 μM of each individual sequence assuming equal proportion of concatenated nucleotide species at the degenerate position during oligonucleotide synthesis

*** that is 0.125 μM of each individual sequence

### Detection of mycoplasma 16S rDNA using m16S_qPCR according to the rules of good laboratory practice MIQE guidelines [13–15] (protocol at a glance)

Critical step: Use Low Binding filter tips and tubes to maximize sample recovery and avoid DNA cross-contamination.

#### DNA extraction from samples

- Add 10 μL of p_GFP (10 pg/μL) as DNA loading tracer to 1 mL of cell-free supernatant sample into 1.5 mL Low Binding conical tube, and vortex.
- Centrifuge at 8,000 g exactly for 5 min at RT.
- Carefully discard the supernatant to spare the very small pellet (maybe difficult to see)
- Lyse the pellet using 180 μL of Lysis Buffer T1 from the NucleoSpin Tissue kit (Macherey-Nagel, cat. No. 740952) by repeating pipetting.
- *In the case of BSL3 or BSL4 samples*, *transfer the supernatant into a new 1*.*5 mL tube*.
- Add 25 μl of Proteinase K and vortex vigorously.
- Incubate at 56°C for at least one hour with frequent vortexing.
- *In the case of BSL3 or BSL4 samples*, *transfer the supernatant into a new 1*.*5 mL tube*.
- Add 200 μL of Lysis Buffer B3 and vortex vigorously.
- Incubate at 96°C for 15 min.
- Vortex briefly.
- *In the case of BSL3 or BSL4 samples*, *transfer the supernatant into a new 1*.*5 mL tube*. *At this stage*, *the tube is ready to exit the BSL3 or BSL4 containment according to local biosafety regulations*.
- Add 210 μL of absolute ethanol and vortex vigorously. White filaments may appear.
- Transfer the whole sample (~500 μL including white filaments) onto a NucleoSpin Tissue column above a collector tube.

*For the next steps the following alternative procedure given by manufacturer has been selected*.

- Centrifuge at 11,000 g for 1 min at RT.
- Discard the flow through solution from the collecting tube.
- Add 500 μL of Wash Buffer B5 on the column.
- Centrifuge at 11,000 g for 1 min at RT.
- Discard the flow through solution from the collecting tube.
- Add 600 μL of Wash Buffer B5 on the column.
- Centrifuge at 11,000 g for 1 min at RT.
- Discard the flow through solution from the collecting tube.
- Centrifuge at 11,000 g for 1 min at RT and throw the collector tube.
- Put the column on the top of a 1.5 mL Low Binding conical tube.
- Add 50 μL of Elution Buffer BE heated to 70°C on the column and incubate for 3 min at RT.
- Centrifuge at 11,000 g for 1 min at RT.
- Add 50 μL of Elution Buffer BE heated to 70°C on the column and incubate for 3 min at RT.
- Centrifuge at 11,000 g for 1 min at RT.
- *If not yet done*, *at this stage the conical tube containing 100* μL of purified DNA *is ready to exit the BSL3 or BSL4 containment according to the local biosafety regulations*
- Aliquot the DNA solution in four 0.5 mL Low Binding conical tubes (25 μL/tube) and kept them frozen at -20°C until use.
- Discard the flow through solution from collecting tube.

#### Real-time PCR of GFP DNA loading probe

- **Critical point:** Wear gloves and use an UV hood#1 solely dedicated to “master mix” for PCR. Treat all the material with UV for 10 min before starting. Do not stock forward and reverse primers as a mixture.

Carry out a master mix with primers "GFP" by adding reagents as shown in Table 3. Vortex the solution and centrifuge for foamless solutions. This GFP mixed master (Green Fluorescence Protein) allows normalization of results by a GFP plasmid DNA reference.

**Table 3:** Master mix GFP qPCR components.

Master Mix GFP	Final Concentration	Volume per reaction tube (μL)
**Sterile nuclease-free water (pH 7/8)**		4.6
**FastStart SYBR Green Master (ROX) 2X (Roche, cat.no. 4913850001)**	1X	10
**Primer GFP_for (20 μM)**	0.2 μM	0.2
**Primer GFP_rev (20 μM)**	0.2 μM	0.2

(see Table 2 for primer sequences)

#### Real-time PCR of mycoplasma 16S rDNA using U1/U8 universal degenerate primers (m16S_qPCR)

- The qPCR is performed as described above for GFP DNA except for the composition of the SYBR Green/Primer mix.

Prepare the master mix for the primers "m16S" by addition of reagents as shown in Table 4. Vortex the solution and centrifuge for foamless solutions. m16S targets 1.5 kb of the 16S ribosomal DNA of *Mollicutes*.

**Table 4:** Master mix m16S qPCR components.

Master Mix m16S	Final Concentration	Volume per reaction tube (μL)
**Sterile nuclease-free water (pH 7/8)**		2
**FastStart SYBR Green Master (ROX) 2X (Roche, cat.no. 4913850001)**	1X	10
**Primer U1 (20 μM)**	1 μM	1
**Primer U8 (20 μM)**	2 μM	2

(see Table 2 for primer sequences)

- Vortex, then shortly centrifuge the SYBR Green/Primer mix to eliminate any foam.
- **Critical point:** Wear gloves and use an UV hood#2 solely dedicated to “prepare qPCR plate”. Treat all the material with UV for 10 min before starting. Do not stock forward and reverse primers as a mixture.
- Add 15 μL of master mix “GFP” or “m16S to each well of a MicroAmp Fast optical 96-well Reaction Plate (Applied Biosystems, cat. no. 4346906).
- Add in duplicate 5 μL of either water, or p_GFP (at 1 ng/mL) or p_m16S (at 1 ng/mL) or samples.
- Carefully close each well by covering the plate with one MicroAmp Optical adhesive Film.
- Centrifuge shortly the plate for 10 s to bring mixtures at the bottom of the well.
- Run the qPCR according to Table 5 in a StepOne Plus (Applied Biosystems) with standard low ramping (3 hours to complete a run).

**Table 5:** Run program steps for the detection of 16S rDNA of mollicutes adapted from [5].

Cycle Number	Step	Temperature	Period	
**1 cycle**	Activation of the enzyme	95°C	10 min	
**40 cycles**	Denaturation	95°C	15 s	
	Hybridation / Elongation	65°C	2 min	Data collection

Data are collected and curves are generated with StepOne software (Applied). Each reaction is carried out in duplicate. The analysis of amplicon strand dissociation is performed at the end of the run to visualize amplification specificity.

To optimize the melting curve, the fluorescence is acquired at every 0.3°C during a 65°C to 95°C temperature gradient.

- Quantification cycle (Cq) method determination: manually set the threshold above the background on the lower limit of exponential phase of kinetic amplification. Cq is the crossing point between threshold and kinetic.

**Fig 1:**
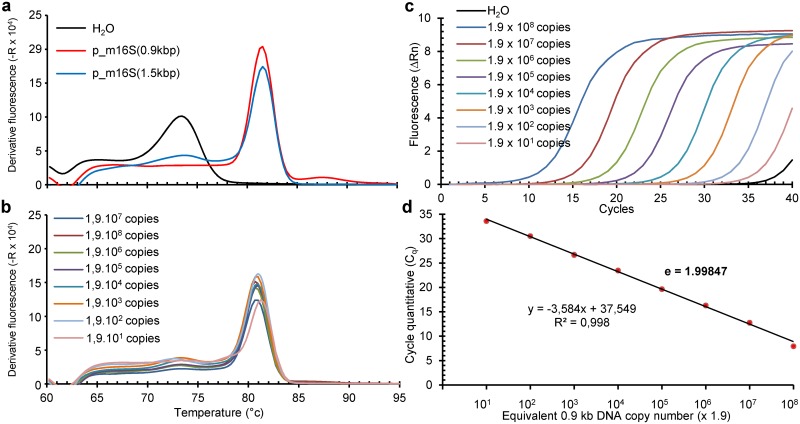
Melting curves and amplification plots of 16S rDNA amplicons resulting from PCR using U1/U8 primers. Melting curves obtained using p_m16S(0.9kb), a plasmid containing internally deleted 16S rDNA from *M*. *capricolum* subsp. *capricolum* strain California Kid (gi_83283139) (**a**), their reproducibility over multiple quantifications (**b**), with the amplification plot (**c**) and the linear regression analysis of Cq as a function of DNA copy input number (**d**, efficacy E_m16S_ = 1.99847). For (**c**) and (**d**), dilutions were done from a freshly prepared 0.9 kb PCR amplicon obtained from 5 pg of p_m16S(0.9kb) using running conditions depicted in Tables 2 and 5 with DNA concentration measured by NanoDrop^™^ (http://www.nanodrop.com/).

In order to determine primers efficiencies, a template qPCR reaction is performed for each primer couple and then diluted to generate linear standard curves. Primer efficiencies are reported in Fig 1.

Relative quantities of DNA copies are calculated using primer efficiency (e) according to the mathematical model of Pfaffl [16].

In the case of doubtful results from the analysis of the melting curve, the qPCR programme is modified by adding a 10% ramping time between denaturation at 95°C and hybridization/elongation time at 65°C. At the end of the PCR, the plate is kept frozen until further use.

#### Size determination of m16S PCR amplicons and sequencing

The amplicons generated during the m16S_qPCR were analysed by electrophoresis in an agarose gel essentially as detailed in [3, 5] except for the use of GelRed (https://www.brunschwig-ch.com/pdf/news/BI_GelRed.pdf) to visualize DNA bands under a UV table equipped with a digital camera.

Amplicons of 1.5 kb size were recovered using NucleoSpin Gel and PCR Clean-up kit (http://www.mn-net.com/Portals/8/attachments/Redakteure_Bio/Protocols/DNA%20clean-up/UM_PCRcleanup_Gelex_NSGelPCR.pdf) according to the manufacturer recommendations and sequenced by Sanger’s method.

#### Calculation of sensitivity and specificity

They were calculated as reported [17, 18]. True positive samples include samples found to be positive in all methods and samples found to be positive with the qPCR method and/or for which the mycoplasma contamination status was known. True negative samples include samples found to be negative in all methods and samples found to be negative by at least qPCR with the exception of three samples for which the very low contamination status was known because of having been intentionally contaminated (i.e. labelled as false negative). Statistical analysis for differences in proportion was performed using Fischer’s test.

## Results and discussion

Because of the need of many colleagues working with cell lines and viruses requiring BSL2 to BSL4 biosafety levels, the aim was to find and to implement a mycoplasma detection assay sensitive enough that it could be used universally. Within CelluloNet BioBank, the indirect Hoechst staining assay based on the human MeWo cell line [6] (ECACC ref. 93082609) as cell indicator and MycoAlert are routinely used. Several intrinsic limitations were encountered. MeWo cells are destroyed by many viruses, thus preventing their use as universal reporter cells. At least one mycoplasma strain (unfortunately not genotyped as it was observed prior to the setting of m16S_qPCR) was found by serendipity to escape detection by MycoAlert. Furthermore adapting the Hoechst staining assay to BSL3 and BSL4 pathogens appears to be very difficult owing to stringent biosafety rules. Testing the suitability of a new method called PlasmoTest based on the detection of mycoplasma lipopeptides was then considered. This assay looks in principle rather easy to perform with pathogenic material since prior to the test, samples should be heat-inactivated for 15 min at 100°C, i.e. a condition where all human viruses are destroyed if one excludes prions. This assay proved to be useful but suffers from limited sensitivity and from relying on maintenance of a reporter cell line. Alternatively, it was reasoned that, theoretically, it might be feasible to implement the universal PCR detection that was developed using universal U1 and U8 degenerate primers targeting the 16S rDNA of *Mollicutes* by Johansson et al. [5] and improve its sensitivity by performing the PCR in real-time. It was first confirmed that all mycoplasma strains with recorded 16S rDNA from GenBank and SILVA databank [19] can be targeted by blasting with U1 and U8 primers. Furthermore a BLAST search in SILVA with up to five mismatchs located upstream to the last 3’ fifth nucleotide of each primer recovered the same set of *Mollicutes* enriched with some related bacteria including cyanobacteria and chloroplasts (S1 Table).

To establish a Real-Time PCR with U1/U8 degenerate primers, the 1.5 kb long 16S rDNA from *M*. *capricolum subsp*. *capricolum strain California Kid* (gi_83283139) by was amplified using U1 and U8 primers and the resulting DNA amplicon was subcloned into a plasmid named p_m16S(1.5kb). From this plasmid, the p_m16S(0.9kb) plasmid was derived by internal deletion within the 16S rDNA. The use of the PCR parameters described in [5] gave unsatisfactory results in qPCR. The hybridization/elongation temperature (see S1 Fig) and the amount and the concentration of U1 and U8 primers were therefore optimised. A satisfactory homogenous dissociation curve clearly different from that obtained with water was obtained. This latter non-specific melting curve reflects the formation of primer dimers in the absence of target DNA likely due to both the high primer concentration and their degeneracy. While primer dimers were detected with a Tm = 74 ± 0.44°C, an m16S rDNA amplicon was found to peak at 80.8 ± 0.15°C (Table 6). When run on p_m16S(0.9kb), the Cq linearly correlated with the copy number of p_m16S(0.9kb) DNA input (Fig 1a–1d). Importantly the melting curves obtained with p_m16S(1.5kb) and p_m16S(0.9kb) peaked at the same Tm despite the 0.6 kb difference in the amplicon size (see below for size confirmation). This optimized method was called “m16S_qPCR”. That a Real-Time PCR of long DNA fragments can be performed with non-degenerate primers has been independently reported to detect two bacterial non-ribosomal genes [20, 21].

**Table 6:** Mean and SD values of C_T_ and Tm from amplification plot and melting curves observed with p_GFP and p_16S(0.9kbp) used as DNA load and 16S positive controls respectively.

Primers	DNA source	Number of measurements	C_T_ (mean ± SD)	Tm (mean ± SD
GFP	water	17	27.7 ± 1.4	76.4 ± 0.26
	p_GFP	15	17.6 ± 1.5	83.5 ± 0.05
16S U1/U8	water	21	34.4 ± 2	74 ± 0.44
	C16S p_m16S(0.9kbp)	18	19 ± 1.6	80.8 ± 0.15

**Fig 2:**
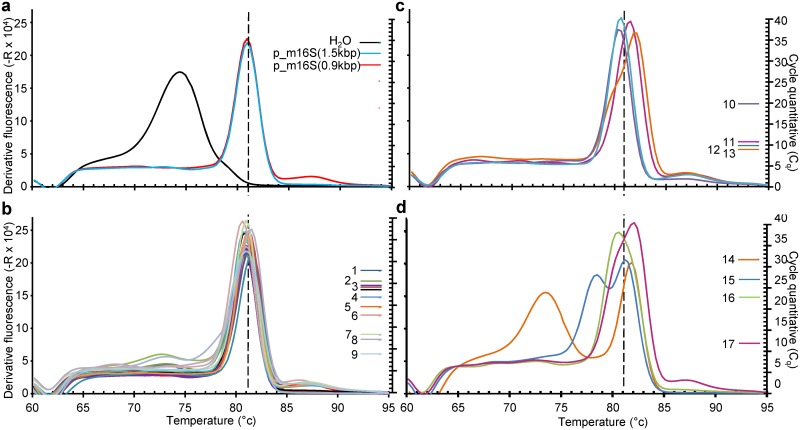
m16S Melting curve (left, curves) and Cq (right, ladder) values obtained with a DNA panel from several mycoplasma species showing diversity of the melting curve from similarity with p_m16S(0.9kb) positive reference (a, b), different Tm (c) and bimodal curves (d). (**b**) ^*1*^ *M*. *imitans;* ^*2*^ *M*. *canis;* ^*3*^ *M*.*arginini*, *M*. *salivarium*, *M*. *alkalescens*, *M*. *agalactiae*, *M*. *canadense*, *M*. *apricolum* subsps. *capricolum;* ^*4*^ *M*. *mycoides* subsp. *capri;* ^*5*^ *M*. *putrefaciens;* ^*6*^ *M*. *verecundum;* ^*7*^ *M*. *moatsii*, ^*8*^ *M*. *fastidiosum;* ^*9*^ *M*. *alvi*. *(****c****)* ^*10*^ *M*. *ovipneumoniae;* ^*11*^ *M*. *iguana;* ^*12*^ *M*. *mycoides* subsp. *mycoides;* ^*13*^ *M*. *opalescens*. *(****d****)* ^*14*^ *M*. *yeatsii;* ^*15*^ *M*. *columborale;* ^*16*^ *M*. *lipofaciens*, ^*17*^ *M*. *fermentans*. Tm peak of 16S rDNA amplicon is indicated by the dotted line on each graph.

The ability of m16S_qPCR to detect and to quantify available DNA stocks from an internal mycoplasma collection was then tested. All samples with measurable DNA contents using Qubit dsDNA HS Assay Kit (ThermoFischer Scientific, cat. No. Q32851), i.e. >0.4 ng/mL, gave a measurable signal. Amplicons from most mycoplasma strain samples gave a melting curve that was identical with that observed with p_m16S(0.9kb) positive control (Fig 2a–2c) peaking at ~81°C. Two other samples gave rise to amplicons exhibiting a small Tm shift (Fig 2c) and/or a double peak (Fig 2d) corresponding in one case of the presence of the primer-dimer peak at ~74°C due to the very low DNA content of this sample.

**Fig 3:**
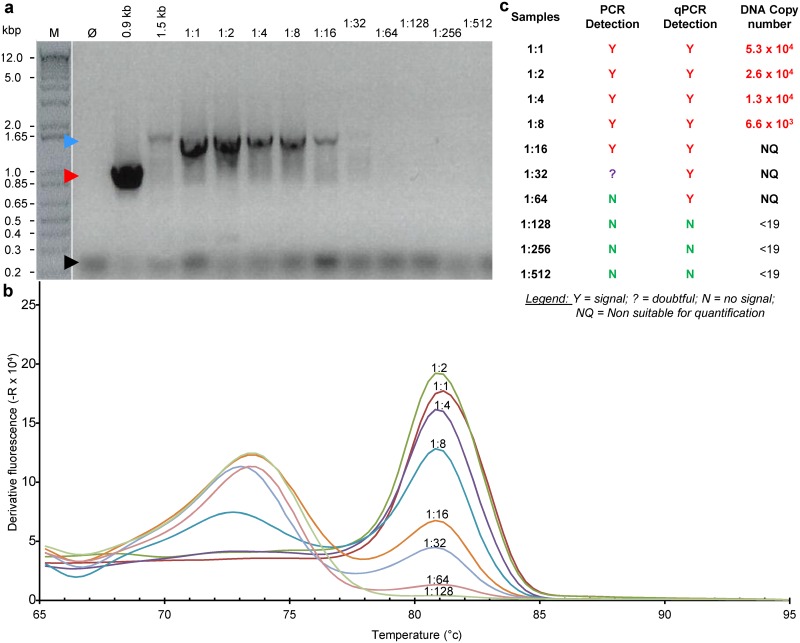
Relative sensitivity of qPCR and PCR using parameters optimized for qPCR. **(a)** PCR product imaging after electrophoresis on agarose gel and staining. **(b)** Melting curves of qPCR samples **(c)** Table summarising data illustrated in (**a**) and (**b**). A cell culture supernatant known to be contaminated by mycoplasma was serially diluted and a sample of each dilution was run on qPCR. At the end of the qPCR run, the obtained qPCR product from each sample was analysed on agarose gel as standard PCR products.

**Fig 4:**
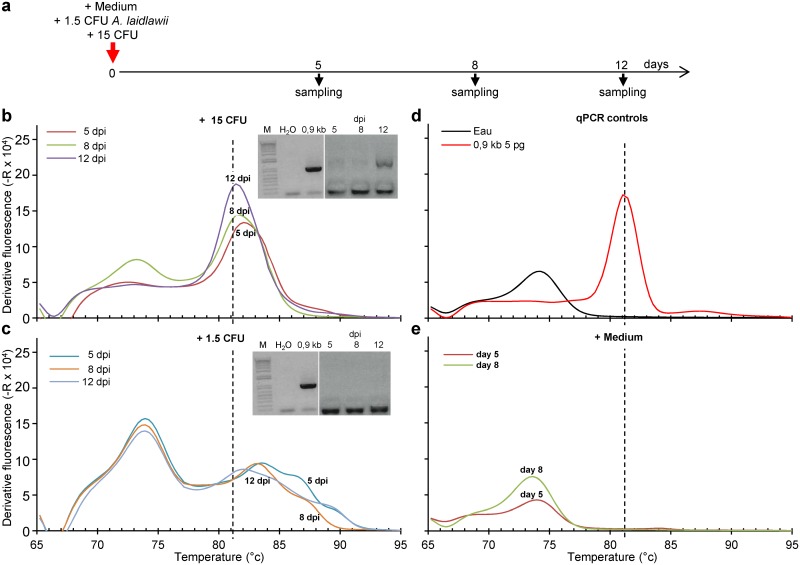
Comparison of the sensitivity of PCR and qPCR assays. MeWo cells (1.5 x 10^6^) seeded one day before in 25 cm^2^ tissue culture flasks were inoculated with either culture medium (Medium) or 1.5 or 15 CFU of *Acholeplasma laidlawii (A*.*l*.*)*. Cell free supernatants were collected after 5, 8 or 12 days of culture (**a**) and DNA was extracted and analysed by qPCR and visualization of PCR products after electrophoresis on agarose gel (**b-d**). (**see also** Table 7).

As the qPCR assay was using the same set of U1/U8 primers previously recommended for detection of mycoplasma [5], the relative sensitivity of the PCR and qPCR assay using the optimised PCR parameters was evaluated by testing serial dilution of a known mycoplasma contaminated cell free supernatant using the qPCR with either visualization of the resulting PCR product after electrophoresis in agarose gel (Fig 3a) or by analysing the final melting curve of the PCR product (Fig 3b). The qPCR was found to be ~4 times more sensitive than the classical PCR. According to this experiment and the sample used, the lowest amounts of genomic DNA copies that could be detected can be estimated to be ~700 genome copies. The sensitivity of PCR and qPCR was further investigated and compared to the indirect Hoechst staining, MycoAlert^™^ and/or PlasmoTest^™^ detection assays. To ensure working with “true” mycoplasma free and contaminated samples, multiple flasks of a mycoplasma-free cell line were intendedly contaminated with either 1.5 or 15 colony forming units (CFU) of *Acholeplasma laidlawii*. *S*amples were harvested after 5, 8 and 12 days of cell culture and tested by every assay (Fig 4a). MycoAlert^™^ and PlasmoTest^™^ were unable to detect mycoplasma in any sample whereas the Hoescht indirect assay indicated the presence of mycoplasma in cells inoculated with 15 CFU after 5, 8 and 12 days of culture and only after 12 days of culture after contamination with 1.5 CFU. A clear 1.5 kb band could be detected only after 12 days of culture of the highest mycoplasma inoculum (Fig 4b and 4c **(insets)** and Table 7). Whichever the duration of the culture and mycoplasma inoculum size, all contaminated samples were successfully detected by analysing the melting curve obtained in qPCR although quantification could be performed only after 12 day amplification in culture of the 15 CFU mycoplasma load (Fig 4, Table 7). If one assumes a constant generation time over the 12 days culture of cells inoculated with 15 CFU leading to 1.49 x 10^5^ genomic copies per mL of the cell-free supernatant, the generation time of *Acholeplasma laidlawii* in cell culture conditions could be estimated to be ~12 hours. This value is much higher than the 1 h generation time reported in optimised medium conditions [22]. While such a 12-fold difference can be due to partial recovery of the mycoplasmas from the cell culture, this suggests that mycoplasma growth in tissue culture may be slowed down possibly due to shortage of cell derived nutriments. From the 12 h generation time and assuming comparable and constant growth after inoculation with only 1.5 CFU of *Acholeplasma laidlawii* and 100% mycoplasma recovery from the flasks, the qPCR sensitivity to detect this mycoplasma strain in cell culture supernatant obtained by melting curve examination appears to be as low as an estimated ~1–2 genomic copies present at 5 d.p.i. after the lowest inoculum of *A*. *laidlawii*, i.e. close to the 19 copies limit that can be quantified in 5 μl of a DNA solution using the p_m16S(0.9kb) reference (see Fig 1).

**Table 7:** Comparison of the sensitivity of five mycoplasma detection assays. MeWo cells were inoculated with either culture medium (Medium) or 1.5 or 15 CFU of *Acholeplasma laidlawii (A*.*l*.*)*. Cell free supernatants were collected after 5, 8 or 12 days of culture and tested with the various assays (see also Fig 4 for details).

Seeding at day 0	Days in culture	MycoAlert^™^	PlasmoTest^™^	Hoechst	PCR	qPCR	
						Detection	Quantification
Medium	5	N	N	N	N	N	NQ
	8	N	N	N	N	N	NQ
*A*.*l*. 1.5 CFU	5	N	N	N	N	Y	NQ
	8	N	N	N	N	Y	NQ
	12	N	N	Y	N	Y	NQ
*A*.*l*. 15 CFU	5	N	N	Y	Trace?	Y	NQ
	8	N	N	Y	Trace?	Y	NQ
	12	N	N	Y	Y	Y	1,29 x 10^5^

**Fig 5:**
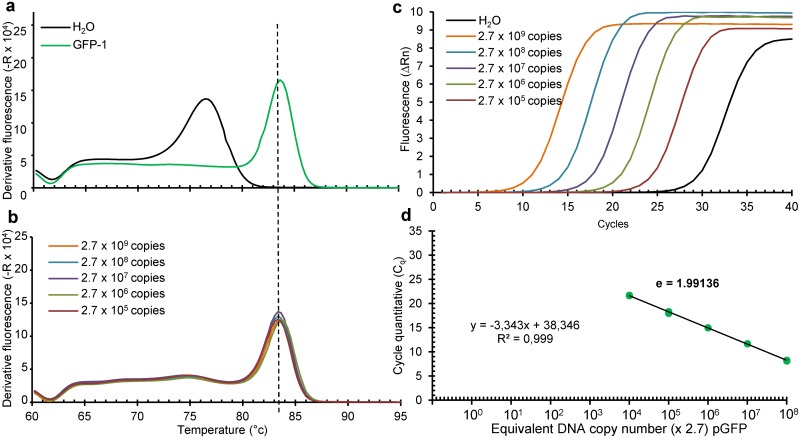
Melting curves and amplification plots of GFP DNA amplicons resulting from PCR using GFP. Melting curves obtained using p_GFP (**a**), their reproducibility over multiple quantification runs (**b**) with the amplification plot (**c**), and the linear regression analysis of Cq as a function of DNA copy input number (**d**, efficacy E_GFP_ = 1.99136) Tm peak of GFP amplicon is indicated by the dotted line. For (**c**) and (**d**), dilutions were done from a freshly prepared 99 bp PCR amplicon obtained from 5 pg of p_GFP using running conditions depicted in Table 2 with DNA concentration measured by NanoDrop^™^ (http://www.nanodrop.com/).

To evaluate to what extent m16S_qPCR can be routinely used to detect mycoplasma contamination of cell cultures and virus stocks, about one hundred cell-free supernatants or virus stocks were analysed and the results were compared with those obtained using the indirect Hoechst staining, MycoAlert and/or PlasmoTest detection assays whenever technically possible. Since the first step of m16S_qPCR required DNA purification from the unknown samples, a small amount of p_GFP was added to each sample prior to DNA extraction. The use of this DNA loading probe allowed us to ensure recovery of DNA free from PCR inhibitors from every sample by running GFP-specific qPCR as illustrated in Fig 5. When run using the m16S_qPCR method no amplicons other than that of the primer dimers were amplified from 5 pg p_GFP plasmid thus validating this DNA loading probe as being neutral (S2 Fig).

**Fig 6:**
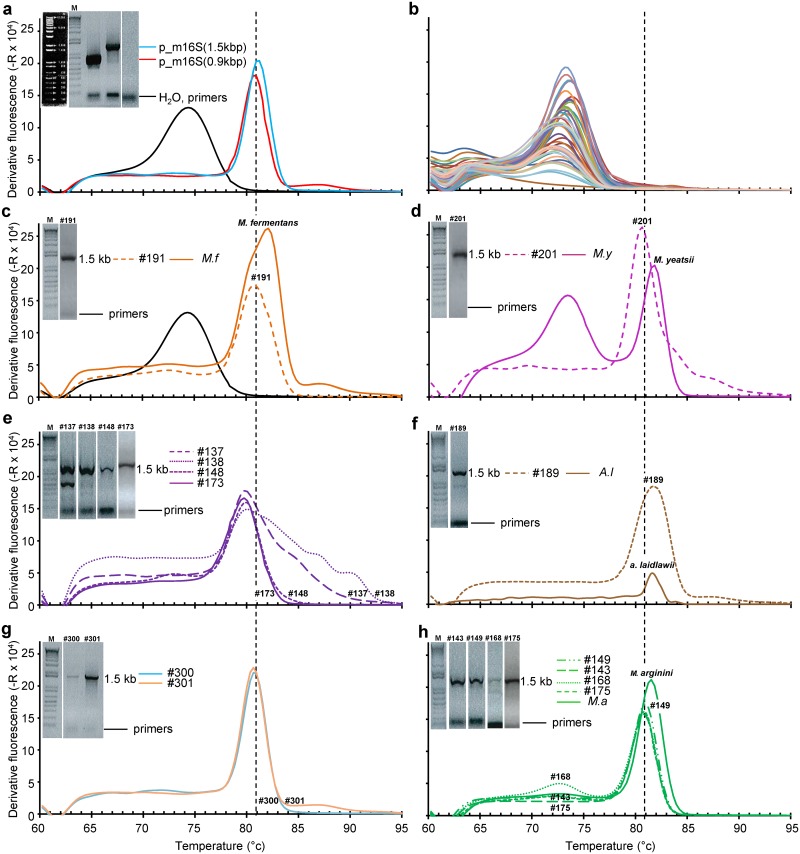
Dissociation curves of m16S qPCR and amplicon size (insets) of DNA samples from cell-free supernatants and/or virus stocks and comparison with DNA from mycoplasma cultures when available with controls (a), samples (# follow by number) without detectable mycoplasma 16S rDNA (b) M. fermentans, (c), sample(s) with contaminated M. yeatsii (d) M. hyorhinis (e), A. laidlawii, (f), M. cottewii (g) and M. arginini (h). For sake of clarity, only corresponding amplicons run on agarose gel electrophoresis are shown in the inset with the 1 kb ladder markers (11, 10, 9, 8, 7, 6, 5, 4, 3, 2, 1.65, 1, 0.85, 0.65, 0.6, 0.4, 0.3, 0.2 and 0.1 kb dsDNA) shown lane M in (**a**). Tm peak of 16S rDNA amplicon is indicated by the dotted line on each graph.

DNA extracted from most of the unknown samples and run using m16S_qPCR showed only the melting curve of the primer dimer (Fig 6a and 6b, see the amplicon sizes in the inset in **a** for water, p_m16S(0.9kb) and p_m16S(1.5kb) controls). Those samples were considered to be below the limit of 19 “16S rDNA” copies/ sample. An m16S rDNA amplicon exhibiting a melting curve peaking at ~81°C was detected in 14 samples (Fig 6c–6h). Amplicon size was determined after agarose electrophoresis (Fig 6, insets) and found each time to be ~1.5 kb long, thus excluding a possible contamination with the 0.9 kb amplicons from the positive p_m16S(0.9kb) reference (Fig 6, compare insets of panels **c-h** with that of panel **a**). Furthermore, sequencing revealed the presence of 6 different mycoplasma strains, namely *M*. *hyorhinis*, *A*. *laidlawii*, *M*. *arginini*, *M*. *fermentans*, *M*. *yeatsii* and *M*. *cottewii* thus further excluding contamination by positive *M*. *capricolum* subsp. *capricolum* strain California Kid derived control. The contamination levels varied from 1.3 x10^3^ and 5.2 x 10^7^ rDNA copies/mL of cell-free supernatant or virus stock, i.e. over a 4 log range. While the first four strains are known to be prevalent in contaminating cells in culture (see [23] and cited references herein), a contamination with *M*. *yeatsii* and *M*. *cottewii* came as a surprise. Indeed, their only known ecosystem is the auditory meatus (or external ear canal) of goats where there are commensal [24, 25] although they have been also found one or twice in bovine milk [26] and bovine lung tissue [27]. A related species have been also recently detected in cultured chicken cells [28].

**Fig 7:**
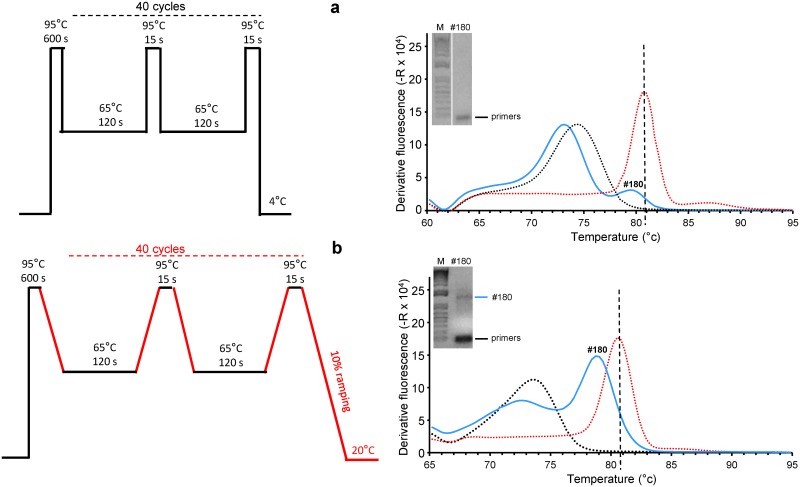
Improvement of mycoplasma DNA detection by slowing the temperature ramping during amplicon melting. Melting curve of sample #180 obtained after 100% ramping (**a**) and 10% ramping (**b**) with amplicon size determination (**a**,**b**, inset). Water and p_m16S(0.9kb) are shown in black and red dotted lines, respectively. Tm peak of 16S rDNA amplicon is indicated by the dotted line on each graph.

In 4 samples, the melting curve profile did show a small shoulder at a higher temperature than that of the melting curve of the primer dimer (Fig 7a). An additional PCR program was then developed, aiming at enhancing the signal that seems to peak around ~81°C in these doubtful samples. This consisted of introducing a 10% ramping for both 65°C to 95°C heating and the 95°C to 65°C cooling phases as shown in Fig 7a and 7b (left). This protocol has the disadvantage of increasing the overall PCR time by 50% but did not change the melting curves of the primer dimer and the positive control (Fig 7, compare right curves in **a** and **b**) In 3 cases, either the 81°C shoulder disappeared or was not notably improved. In one case a peak became clearly visible at ~81°C (Fig 7b). Correlatively, only the primer dimer band was detected in the 3 former samples while a 1.5 kb band could be visualized and sequenced from the fourth sample (Fig 7b, **inset**).

A few other procaryotes can be also detected with the PCR as predicted by blasting U1 and U8 primers against bacterial genome databases (see S2 Table for list). This was experimentally verified from purified DNA from two *Fusobacterium necrophorum strains*, one *Fusobacterium nucleatum strain*, six *Streptococci*, *Listeria monocytogenes* and *Mycobacterium tuberculosis* (S3 Fig compare **b-d** with **a**, see also amplicon sizes in **f**). In contrast, and in agreement with prediction from blast studies, several other bacteria families were found to escape detection (S3e Fig). The detection of some bacteria other than mycoplasma in cell-free supernatants or virus stocks is an advantage since this will warn of a possibly latent bacterial contamination that is as undesirable as is a mycoplasma contamination. As a matter of fact, an accidental bacterial contamination was once detected by the 16S qPCR before it could be seen under the microscope in a tissue culture sample. The U1/U8 primer pair is however unsuitable for the detection of phytoplasma because of a relative abundance of chloroplast DNA that is also targeted by these primers (see S1 Table and S4 Fig). This point has been blindly verified on 12 plant DNA samples contaminated or not with phytoplasma and kindly provided by Nicolas Sauvion (INRA, Montpellier, France).

Among 87 cell-free supernatant or virus stock samples tested, ~17% were found positive for mycoplasma contamination by m16S_qPCR. A few samples (4.6%) could not be tested by any of the other three methods because of technical constraints. From the qPCR positive samples (that were also tested with one, two or three of the other assays, 4.6% were also detected as being contaminated by mycoplasma and 8.05% escaped detection by one or two of other assays. Furthermore, 8 samples in which no 16S rDNA could be detected by m16S qPCR were found to give positive signals by one assay (or even by 3 different assays for one sample) and 2 with doubtful results for at least one assay (11.5%). The 10 samples giving just above threshold signals by MycoAlert could not be confirmed using this assay upon testing of cells that have been infected by these viral stocks and it was speculated that concentrated stocks of enveloped viruses may contain a cell-derived enzymatic source of ATP resulting in possible signal bias using this test.

**Fig 8:**
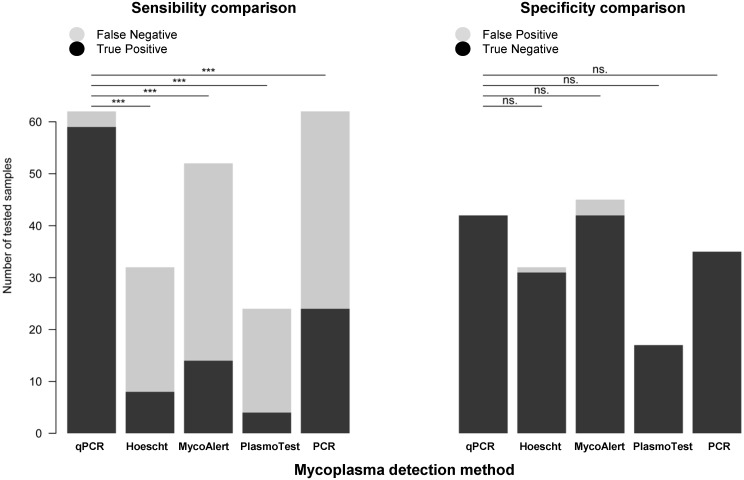
Sensitivity and specificity of five mycoplasma detection methods. The sensitivity of the qPCR test was significantly better than the four other tests (***, p<6.5 x 10^−12^ and below, Fischer’s test.). The specificity levels of all tests did not statistically differ (n.s., p>0.24 and above, Fischer’s test). See also material and methods section for details.

By taking into account the samples tested with their mycoplasma positive or negative status, the sensitivity and specificity were independently calculated for each of the five methods used to detect mycoplasma (Fig 8). The sensitivity of qPCR, Hoechst, MycoAlert, PlasmoTest and PCR is 95.2%, 25.0%, 26.9%,16.7% and 38.7%, respectively, with statistical analysis revealing a significant superiority of the qPCR over all other methods at p<6.5 x 10^−12^ and below. The specificity is very good (98.8%-100%) for all tests. Importantly, those figures are relative to each other and their absolute values will be known only by retrospective analyses of much larger sampling size made by several independent laboratories.

**Fig 9:**
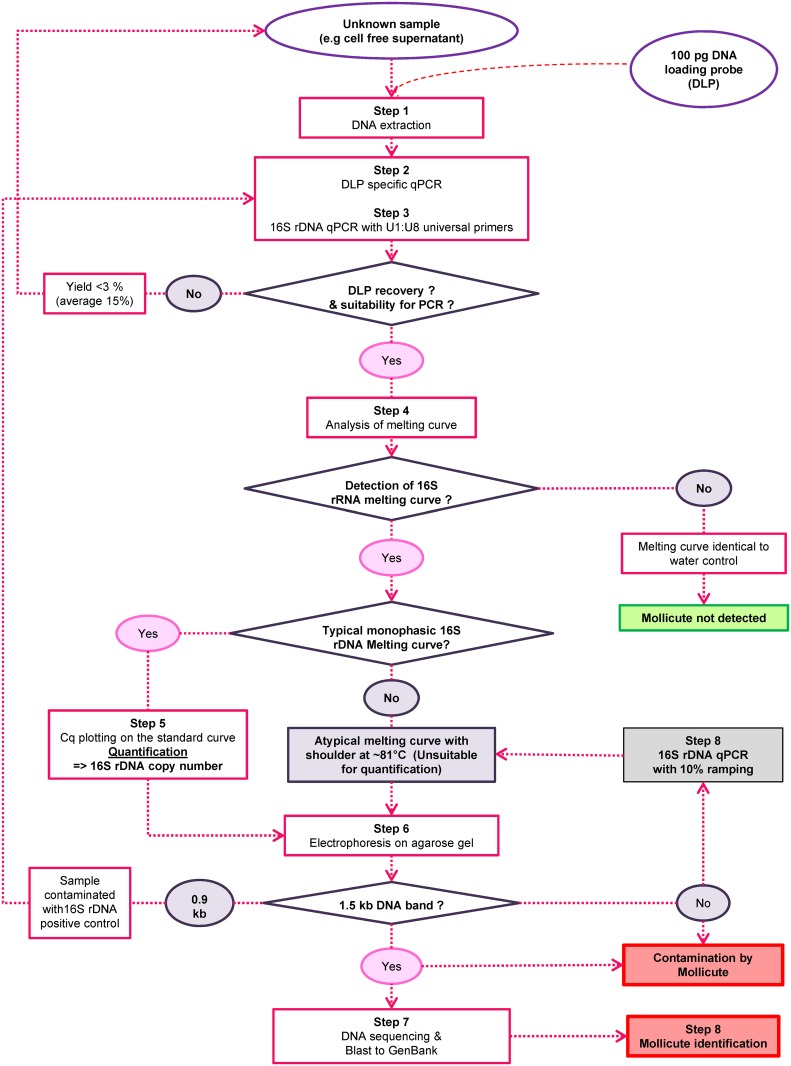
Workflow diagram of decision making.

In conclusion, the m16S_qPCR method to track contamination of cultured cells and cell derived products associates high sensitivity, a very broad range covering the entire *Mollicute* class and usefulness in controlling the absence of mycoplasma contamination of viral stocks requiring any level of biosafety containment. In addition it includes an internal DNA loading probe and a positive reference that is easily tracked in case of accidental contamination of the samples by this reference. A decision-making chart-flow has been built and adopted (Fig 9):

Step 1: A known amount of p_GFP as DNA loading probe is added to cell-free samples to be tested and DNA is purified.
Step 2: The efficiency of DNA purification and the absence of PCR inhibitors is determined by GFP-specific qPCR. In case of low or abnormal GFP signal, DNA purification is performed again.
Step 3: m16S-qPCR is run on the DNA sample, water as negative control and p_m16S(0.9kb) as reference positive DNA.
Step 4: Amplicon melting curve is analysed.
  - 4(a) a melt curve identical to primer indicates lack of detectable mycoplasma contamination (<19 copies/sample) and no further analysis is required.
  - 4(b) an atypical melt curve with a visible shoulder peaking around 81°C that suggests a 16S rDNA signal, go to step 6.*
  - 4(c) a melt curve nearly identical to that obtained with p_m16S(0.9kb) positive control indicates mycoplasma contamination; go to step 5 for quantification.
Step 5: Cq plotting on standard curve gives the contamination level
Step 6: Check amplicon size by agarose gel electrophoresis
  - 6(a) 1.5 kb amplicon size: mycoplasma (or bacteria) contamination is confirmed. Go to step 7.
  - 6(b) no signal corresponding to a 1.5 kb amplicon size with small Tm shoulder at ~81°C indicates a low mycoplasma contamination.
  - 6(c) 0.9 kb amplicon size: accidental contamination with 16S rDNA standard: go to Step 1 to run again the sample.
Step 7: Amplicon can be sequenced for identification of the prokaryote contamination.
  * Note in case of a very low shoulder with a Tm ~81°C the m16S-qPCR can be run again on the sample but using the 10% ramping protocol (Step 8).

The universal m16S_qPCR procedure has several advantages over published available methods including those that are also based on PCR or multi-primer qPCR [4] that detect only subsets of *Mycoplasma* strains, by associating a very broad coverage of the *Mollicute*s and other phylogenetically related bacteria thanks to the use of validated U1/U8 universal primers [5] (our results and see S1 and S2 Tables), a high sensitivity (>19 16S rDNA copies), the incorporation of a DNA loading probe, the use of positive reference control that is traceable and the possibility of strain identification to help find the origin of a contamination. Its usefulness and accuracy on cell free supernatants and stocks of viruses that require BSL2 to BSL4 containment was established here. The use of this method by many other laboratories is expected to confirm its potential usefulness as a much needed gold standard to ensure the lack of mycoplasma contamination in every cell culture usage from research in cell biology [1, 3, 29, 30] and virology [31] to clinical use of living cells (grafts with heterologous or autologous cells modified and amplified in vitro (such as in vitro maturated and antigen loaded self-dendritic cells and progenitors expressing a therapeutic gene) or cell derived products such as vaccines, therapeutic antibodies, growth factors and cytokines, oncolytic viruses, viral vectors for gene delivery [32, 33], see OIE regulations (http://www.oie.int/fileadmin/Home/fr/Health_standards/tahm/1.01.07_TESTS_FOR_STERILITY.pdf) United States Pharmacopoeia 63 Regulation (http://assets.sial.com/deepweb/assets/bioreliance/marketing/documents/pdf/h/r/bioreliance_pdfs/O0660810USP63WhitePaperFHR/O0660810USP63WhitePaperFHR.pdf) and European Medicines Agency recommendations (http://www.ema.europa.eu/docs/en_GB/document_library/Scientific_guideline/2013/03/WC500140352.pdf).

## Supporting information

S1 Fig Optimisation of elongation temperature during m16S qPCR of p_m16S(0.9kb), *A*. *laidlawii* and *M*. *pulmonis* DNA. *Tm* peak of 16S rDNA amplicon is indicated by the dotted line on each graph.
(PDF)

S2 Fig Specificity of the m16S_qPCR (a) and GFP_qPCR (b) when tested on the p_GFP DNA loading probe and p_m16S(0.9kb) reference. (PDF)

S3 Fig m16S qPCR detects a subset of bacteria in addition to *Mollicutes* with p_m16S(0.9kb) and water (a), detected (b-d) and non-detected (e) bacteria with amplicons sizes from panels a-d (f). *Tm* peak of 16S rDNA amplicon is indicated by the dotted line on each graph.
(PDF)

S4 Fig Alignments of U1 and U8 primers on plant chloroplast genomic DNAs that were blindly identified after qPCR of phytoplasma free and contaminated plant samples and sequencing of obtained amplicons. (PDF)

S1 Table List of *Mollicutes* targeted by U1 and U8 primers after BLAST search in GenBank and alignments of targeted sequences. (XLSX)

S2 Table List of *Ternericutes* and *Bacteria* targeted by U1 and U8 primers after a search without mismatches or with up to 5 mismatches located upstream of the last 5 nt at primer 3’ end. (XLSX)
