# Supplementary material for: Assessing mycoplasma contamination of cell cultures by qPCR using a set of universal primer pairs targeting a 1.5 kb fragment of 16S rRNA genes
Source: PLoS One. 2017 Feb 22;12(2):e0172358. doi: 10.1371/journal.pone.0172358 (PMC5321415; doi:10.1371/journal.pone.0172358)
Supplement: S1 Fig — Tm peak of 16S rDNA amplicon is indicated by the dotted line on each graph. (PDF) [file pone.0172358.s001.pdf]

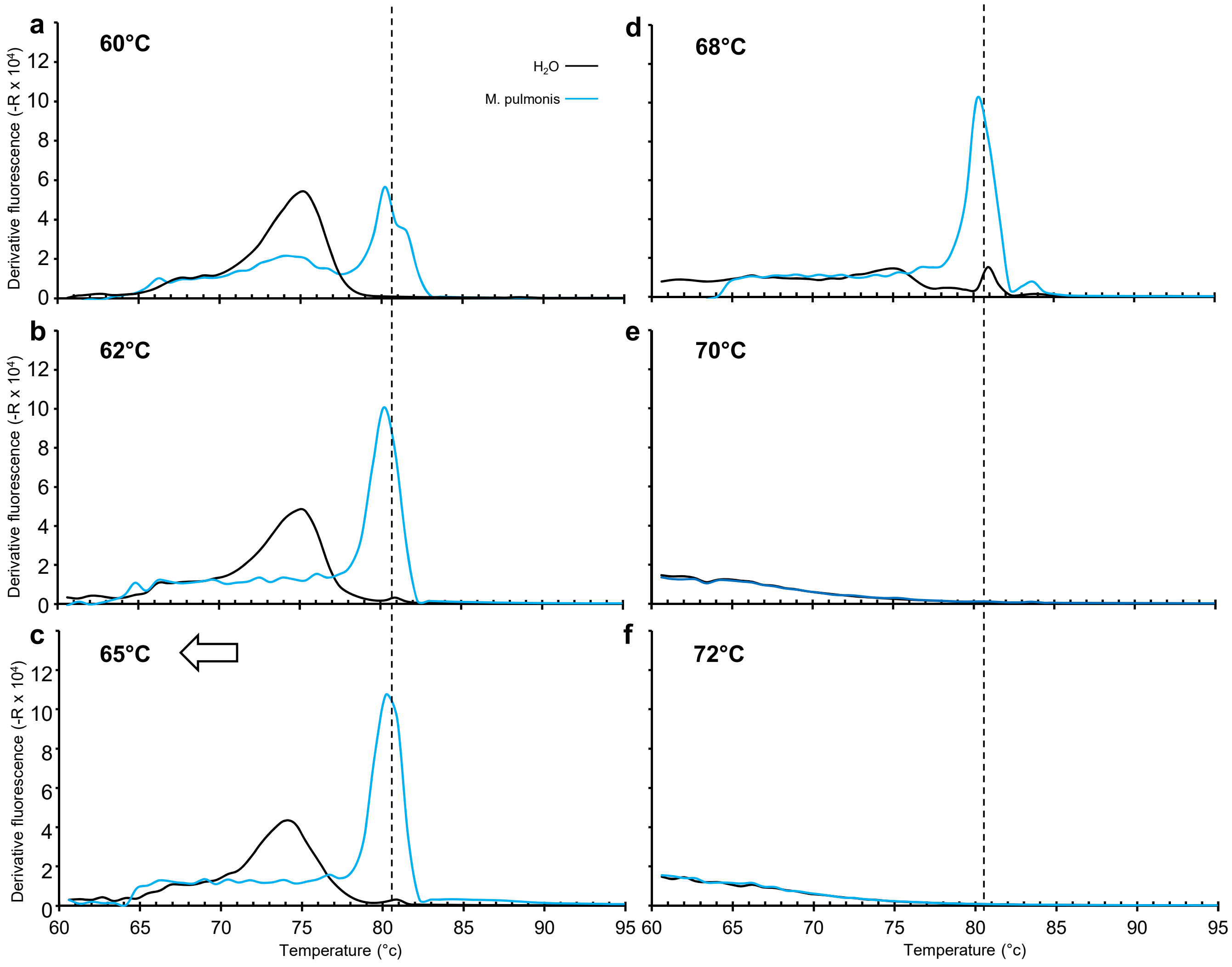

Figure S1: Optimisation of elongation temperature during m16S qPCR of p_m16S(0.9kb), *A. laidlawii* and *M. pulmonis* DNA. *Tm* peak of 16S rDNA amplicon is indicated by the dotted line on each graph.
