# Supplementary material for: Assessing mycoplasma contamination of cell cultures by qPCR using a set of universal primer pairs targeting a 1.5 kb fragment of 16S rRNA genes
Source: PLoS One. 2017 Feb 22;12(2):e0172358. doi: 10.1371/journal.pone.0172358 (PMC5321415; doi:10.1371/journal.pone.0172358)
Supplement: S2 Fig — (PDF) [file pone.0172358.s002.pdf]

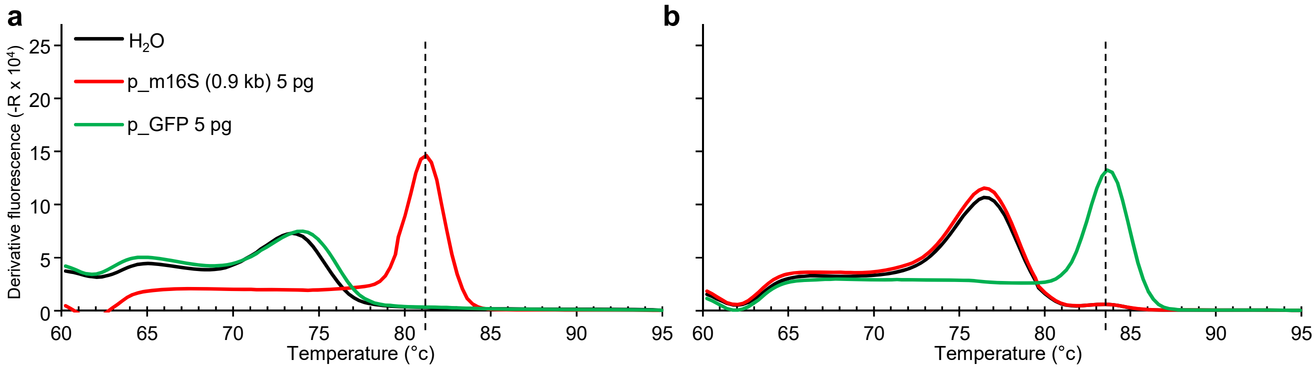

Figure S2: Specificity of the m16S_qPCR (a) and GFP_qPCR (b) when tested on the p_GFP DNA loading probe and p_m16S(0.9kb) reference.
