# Supplementary material for: Assessing mycoplasma contamination of cell cultures by qPCR using a set of universal primer pairs targeting a 1.5 kb fragment of 16S rRNA genes
Source: PLoS One. 2017 Feb 22;12(2):e0172358. doi: 10.1371/journal.pone.0172358 (PMC5321415; doi:10.1371/journal.pone.0172358)
Supplement: S3 Fig — Tm peak of 16S rDNA amplicon is indicated by the dotted line on each graph. (PDF) [file pone.0172358.s003.pdf]

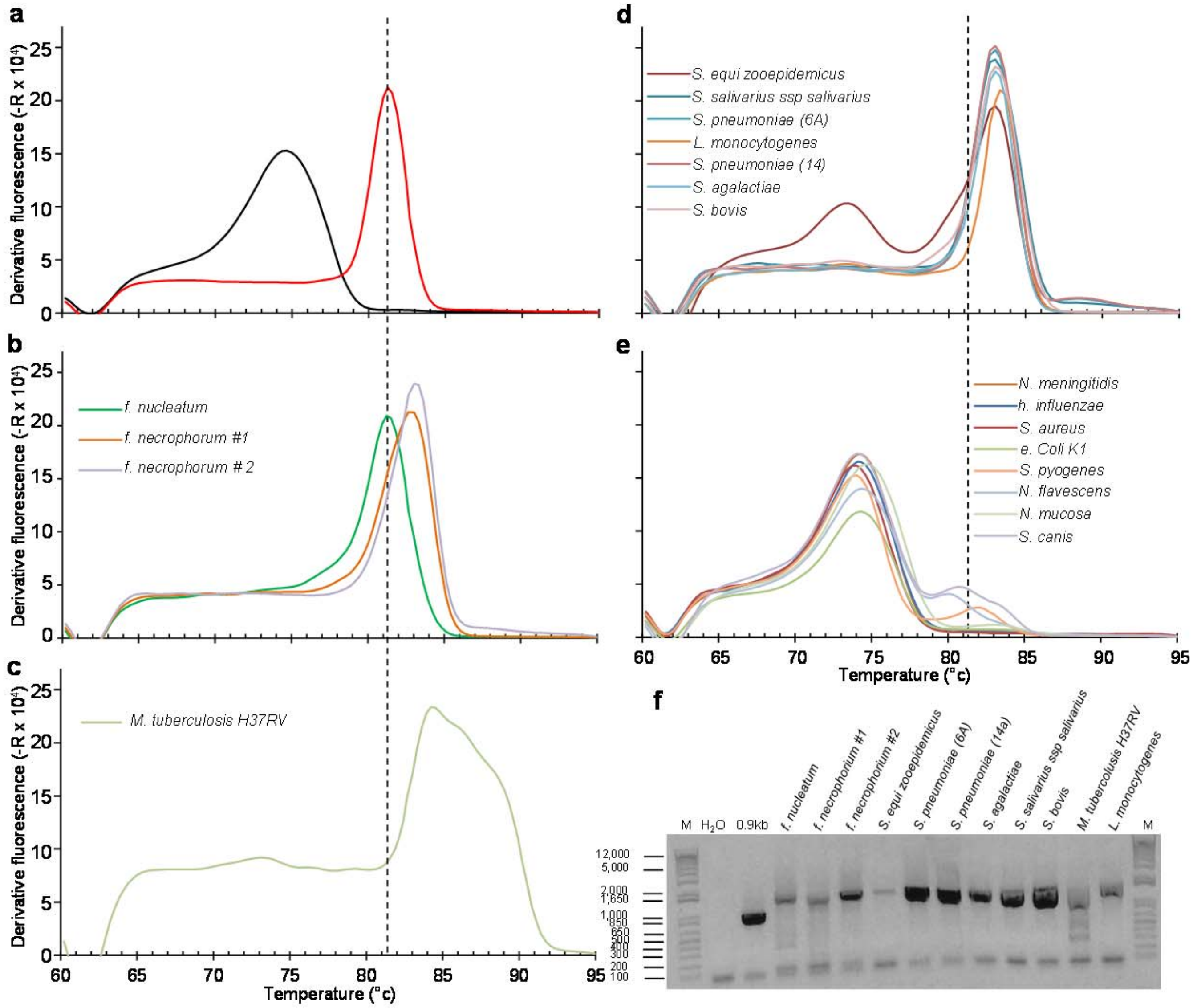

Figure S3: m16S qPCR detects a subset of bacteria in addition to *Mollicutes* with p_m16S(0.9kb) and water (**a**), detected (**b-d**) and non-detected (**e**) bacteria with amplicons sizes from panels **a-d** (**f**). *Tm* peak of 16S rDNA amplicon is indicated by the dotted line on each graph.
