# Supplementary material for: Assessing mycoplasma contamination of cell cultures by qPCR using a set of universal primer pairs targeting a 1.5 kb fragment of 16S rRNA genes
Source: PLoS One. 2017 Feb 22;12(2):e0172358. doi: 10.1371/journal.pone.0172358 (PMC5321415; doi:10.1371/journal.pone.0172358)
Supplement: S4 Fig — (PDF) [file pone.0172358.s004.pdf]

A **Alignments of chloroplast 16S (nt 5-164) with myco16S U1 primer**

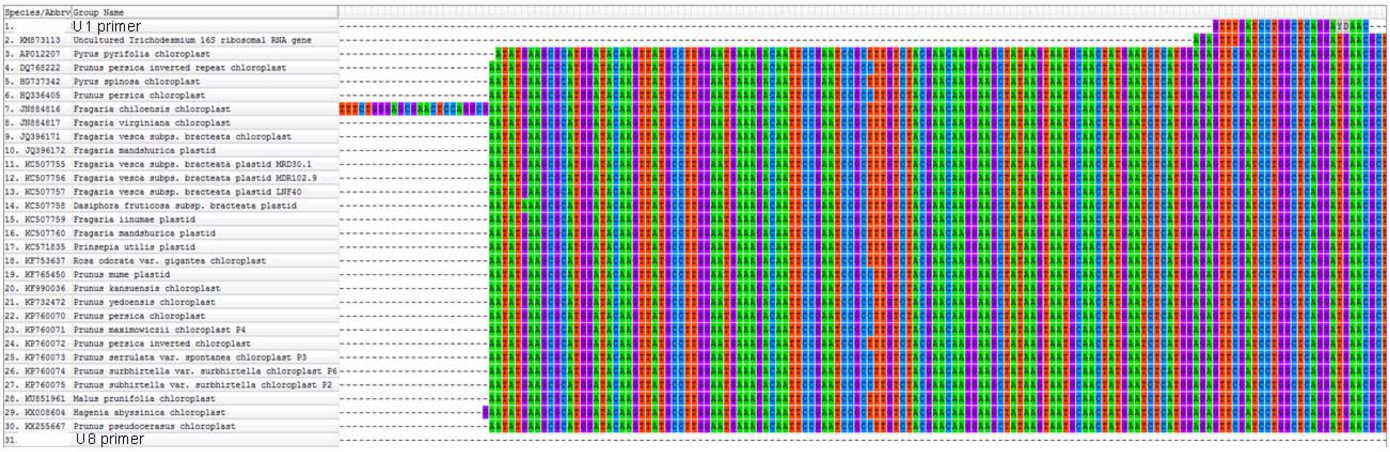

B **Alignments of chloroplast 16S (nt 1575-1724 with myco16S U8 primer**

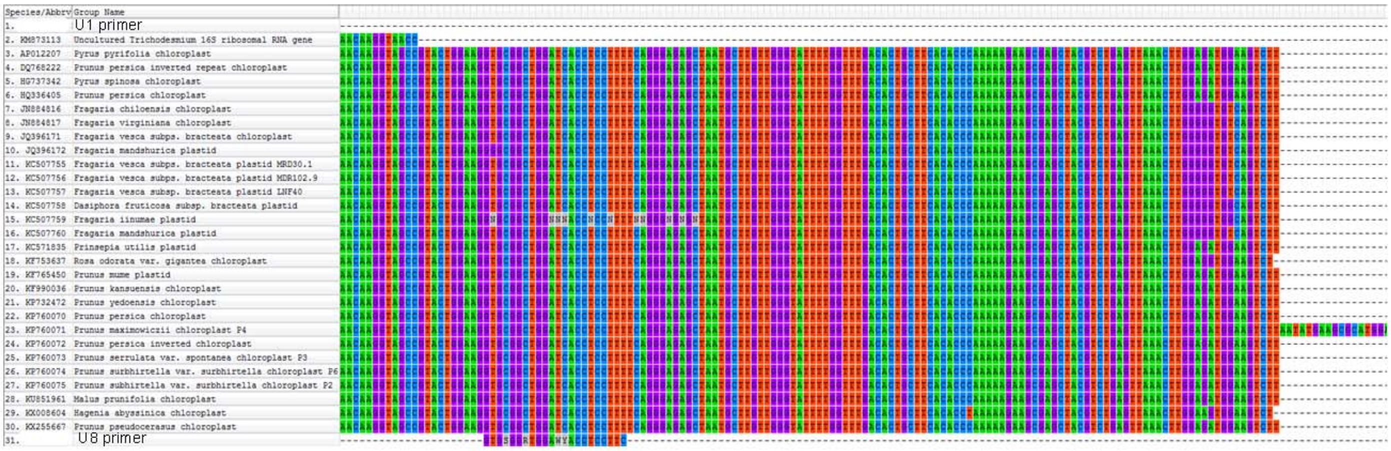

Figure S4. Alignments of U1 and U8 primers on plant chloroplast genomic DNAs that were blindly identified after qPCR of phytoplasma free and contaminated plant samples and sequencing of obtained amplicons.
